# Impact of migration origin on individual protection strategies against sexual transmission of HIV in Paris metropolitan area, SIRS cohort study, France

**DOI:** 10.1186/s12889-015-2051-4

**Published:** 2015-08-20

**Authors:** Thomas Kesteman, Annabelle Lapostolle, Dominique Costagliola, Véronique Massari, Pierre Chauvin

**Affiliations:** Department of Social Epidemiology, Sorbonne Universités, UPMC Univ Paris 06, INSERM, Institut Pierre Louis d’Epidémiologie et de Santé Publique (IPLESP UMRS 1136), F75013 Paris, France; Institut Pasteur de Madagascar, BP 1274 Avaradoha, Antananarivo, 101 Madagascar

## Abstract

**Background:**

The impact of migration and country or region of origin on sexual behaviours and prevention of the sexual transmission of HIV has been scarcely studied in France. The objective of this study was to evaluate if and how individual attitudes of prevention towards HIV infection are different according to country or region of origins in Paris area, France.

**Methods:**

3006 individuals were interviewed in the Paris metropolitan area in 2010. Outcome variables were (i) the intention of the individual to protect oneself against HIV, and (ii) the adoption of a condom-based approach for protection against HIV. To explore factors associated with these outcomes, we constructed multivariate logistic regression models, first taking into account only demographic variables –including country of origin-, then successively adding socioeconomic variables and variables related to sexual behaviour and HIV perception and prevention behaviour.

**Results:**

French and foreign people who have origins in Sub-Saharan Africa declared more intentions to protect themselves than French people with French parents (in foreign men, aOR = 3.43 [1.66–7.13]; in foreign women, aOR = 2.94 [1.65–5.23]), but did not declare more recourse to a condom-based approach for protection against HIV (in foreign men, aOR = 1.38 [0.38–4.93]; in foreign women, aOR = 0.93 [0.40–2.18]). Conversely, foreign women and French women from foreign origin, especially from Maghreb (Northern Africa), reported less intention of protection than French women with French parents.

**Conclusions:**

These results underline the importance of taking culture and origins of target populations into consideration when designing information, education and communication about HIV and sexually transmitted diseases. These results also draw attention to fractions of the general population that could escape from prevention messages.

**Electronic supplementary material:**

The online version of this article (doi:10.1186/s12889-015-2051-4) contains supplementary material, which is available to authorized users.

## Background

In our globalized world, it is now well known that health impacts migration and reversely, migration impacts health [1, 2]. Similarly, ethnicity or geographical origins influence health and health behaviours [3], and more specifically, migration and origins affect sexual knowledge and behaviours [4–6]. In France, immigrants come principally from Southern and Eastern Europe and from former French colonies, mainly Maghreb (Northern Africa) and Sub-Saharan Africa (SSA) [7, 8], including therefore a significant proportion of people originating from countries with high HIV prevalence [9]. Also, in France as in many other European countries, immigrants are disproportionally represented among the poor and the socially excluded populations [10]; which can make them highly vulnerable to HIV/AIDS [9, 11]. Among this migrant population, an important part live in Paris and its suburbs (the capital region), where they now account for 20.2 % of the population [8]. In France in 2010, 57.2 % of new HIV diagnoses were due to heterosexual transmission and 47.7 % occurred in people born abroad [12]. Although the proportion of people born abroad among new HIV diagnoses decreased during the last decade in France, migrants are still disproportionately affected by the epidemic, in particular people from SSA who accounted for 31.7 % of new HIV diagnoses in France in 2010 [12]. Since 2001, migrants are considered as a priority for control programs by the French health authorities [13, 14], and targeted prevention interventions have been conducted [15–17], but few or none has been reported in the scientific English literature [11].

Prevention practices against the HIV infection are, at least partially, driven by intended or unconscious attitudes that take into account individuals’ health knowledge as well as their social values [18–20] and both of them are different according to country of origin [20]. More generally, the social determinants of these practices include people’s demographics (sex, age, country of origin), their relationship characteristics (duration, gender values, communication, sexual practice), their social norms (which varied according to their degree of integration into social groups), and the social context of a given intercourse [4–6, 18, 19, 21, 22]. In France, the relationship between country of origin and HIV protection attitudes and behaviours has been scarcely statistically studied [22], partly because of the existing legal restrictions in collecting and processing data regarding ethnicity or migration status [1, 23–27].

The aim of this study was to evaluate whether or not individual attitudes of prevention are different according to country of origin in the Paris metropolitan area. More specifically, our objectives were to investigate whether relationships exist between people’s origin and (*i*) their intention of protection against HIV, and (*ii*) their adoption of a condom-based approach for HIV protection.

## Methods

### Study sample

The SIRS cohort (a French acronym for Health, Inequalities and Social Ruptures) is the first large, representative, population-based French cohort set up to study the social determinants of health and health-care utilization in the field of social epidemiology [28–31]. In 2005, at inclusion, our study population was a multistage-random sample of the adult French-speaking population living in the Paris metropolitan area (Paris and the 3 adjacent departments, a region of 8.7 million inhabitants). The primary sampling units were census blocks of about 2000 inhabitants each: 50 of them were randomly selected from the 2595 eligible census blocks in Paris and its suburbs, according to their socioeconomic situation. Sixty households were then chosen at random in each census block. Lastly, one adult in each household was randomly selected using the next-birthday method and face-to-face interviewed, using a questionnaire that gathers more than 400 variables related to economic status, social integration, health, health-related behaviours, and the use of the health-care system. This cohort study had received ethical and legal authorization from two national authorities for non-biomedical researches: the *Comité consultatif sur le traitement de l’information en matière de recherche dans le domaine de la santé* (CCTIRS) and the *Commission nationale de l’informatique et des libertés* (CNIL).

In 2010, 47.2 % of participants included in 2005 were interviewed again [32]; 0.6 % had died, 1.5 % were too sick to participate, 3.6 % had moved outside the selected census blocks, 8.5 % were not present at the time of the survey, 15.7 % refused to participate again and 22.9 % were lost to follow-up without information. People who could not be re-interviewed in 2010 were replaced by other people of the same census block, following a random methodology identical to the one described above. As in 2005, refusal rate in 2010 was 29.1 %. Sex ratio and mean age of people followed up were not different from newly included people. This study analyzed the data collected in of 2010 among the final sample of 3006 individuals.

### Variables

#### Intention of protection against HIV and declaration of a condom-based approach for HIV protection

Two outcomes have been successively studied. The intention of protection against HIV corresponds to the positive answer to the question “Do you usually do something to protect yourself against HIV?” If yes, the interviewee was then asked “Personally, what do you do to protect yourself against HIV?”; if no, he/she was asked for what reasons he/she did not do so. These two latter questions could be answered the same ways by choosing one or several of the following items: steady use of condoms, abstinence, fidelity, trust, HIV testing, dialog with partner, restricting sexual intercourse to people of the same community, religious faith, none. This questionnaire design allowed us to distinguish, for a given behaviour or attitude (e.g. using condoms), between intended and unintended protective means against HIV infection. We considered that a person adopted a ‘condom-based approach’ when he/she mentioned at least the steady use of condoms as a means of protection against HIV (the term “steady use” being self-defined and self-declared, without any further precision).

#### Migration origin

In this study, we defined participants’ “migration origin” on the basis of both their nationality and their parents’ nationality [33], and we distinguished French people with French parents (the majority of the population) from French people of SSA origin (i.e. French, born to at least one parent with a SSA nationality), foreigners from SSA, French of Maghrebi origin (Maghreb including Mauritania, Morocco, Algeria, Libya, and Tunisia), foreigners from Maghreb, French of European origin, foreigners from Europe, French from other origins, and foreigners from other countries. When parents had different foreign citizenships (in 3.3 % of the cases), the father’s one was the default citizenship considered. All the different categories of French people with foreign origins may include both people born French from foreign parents, and people born foreigner but who subsequently acquired the French citizenship (whatever their country of birth was – in France or abroad - in both cases); making the simplifying assumption that all these situations correspond to a similar level of acculturation [4].

#### Covariables

The association between migration origin and each of our two outcomes was estimated, first only adjusted on age and, then, when further taking into account some behavioural and social characteristics that could play the role of effect modifiers or intermediary factors (Table 1): socioeconomic factors (education and income), religious practice, variables related to sexual biography (couple status, lifetime number of couple relationships, number of sexual partners in the last 5 years, self-perceived sexual orientation), variables related to communication about sexuality and perception of HIV (intimate social network, perceived susceptibility to infection by HIV, declared stigma toward HIV+ people), and variables related to HIV prevention behaviours (use of condoms, voluntary HIV testing). All covariables were tested for univariate association with outcome variables.

**Table 1:** Prevalence of the intention of protection against HIV and the adoption of a condom-based approach for protection against HIV by subgroups. Paris metropolitan area, France, 2010

		Intention of protection against HIV				Adoption of a condom-based approach			
Variables	Category	Men	%	Women	%	Men	%	Women	%
Demographic variables									
Migration origin	French with French parents	372/939	39.6	315/1030	30.6	330/939	35.1	220/1030	21.4
	French of Sub-Saharan origin	34/58	58.3	21/55	38.6	24/58	41.0	14/55	24.8
	Sub-Saharan foreigner	31/45	68.1	28/50	56.2	22/45	48.9	18/50	35.7
	French of Maghrebi origin	34/89	37.9	23/111	20.6	23/89	25.9	14/111	12.9
	Maghrebi foreigner	20/46	43.0	5/41	13.0	14/46	30.4	6/41	14.7
	French of European origin	32/73	43.6	23/110	21.0	29/73	40.4	15/110	14.1
	European foreigner	15/61	24.0	14/60	23.8	14/61	22.7	8/60	13.6
	French of other origin	18/57	32.6	28/95	29.3	12/57	20.9	18/95	18.9
	Other foreigner	13/36	37.0	6/39	15.5	7/36	18.8	7/39	17.2
Age	≤29 years old	233/300	77.8	163/298	54.7	227/300	75.9	137/298	45.9
	30 - 44 years old	203/451	45.1	174/492	35.3	154/451	34.3	113/492	23.0
	45 - 59 years old	91/334	27.4	93/381	24.5	66/334	19.7	57/381	15.1
	≥60 years old	39/318	12.4	33/419	7.9	27/318	8.3	13/419	3.0
Socio-economic variables									
Education level	None or primary school	21/107	19.4	12/114	10.7	12/107	11.4	7/114	6.5
	Secondary school	195/506	38.5	138/578	23.9	167/506	33.0	88/578	15.3
	High school	352/790	44.5	312/898	34.8	295/790	37.4	224/898	25.0
Monthly income per consumption unit	≤1000€	152/283	53.7	112/331	33.9	123/283	43.6	84/331	25.4
	1001 - 1500€	139/312	44.7	118/351	33.7	118/312	37.8	89/351	25.3
	1501 - 2000€	112/259	43.4	87/312	27.8	96/259	37.1	61/312	19.6
	2001 - 3000€	96/283	34.0	109/339	32.1	80/283	28.2	66/339	19.4
	>3000€	67/266	25.3	37/256	14.4	57/266	21.3	20/256	7.9
Religious affiliation and practice	Regular religious practice	69/211	32.5	86/376	22.9	47/211	22.3	54/376	14.4
	Irregular religious practice	95/228	41.4	86/318	27.1	75/228	33.0	65/318	20.4
	Religious affiliation, no practice	148/401	36.8	128/425	30.2	123/401	30.6	80/425	18.8
	Neither practice nor affiliation	257/562	45.6	163/471	34.5	229/562	40.7	121/471	25.7
Variables related to sexual biography									
Couple status	No relationship	219/303	72.4	147/419	35.1	214/303	70.8	126/419	30.0
	Love affair	126/162	77.5	119/186	63.8	115/162	71.1	105/186	56.4
	Non-cohabiting couple	57/88	65.5	36/77	46.2	51/88	58.3	30/77	38.9
	Cohabiting couple	165/851	19.4	162/907	17.8	94/851	11.0	59/907	6.5
Lifetime number of couple relationships	Never any couple relationship	259/323	80.3	151/258	58.3	248/323	76.8	133/258	51.6
	One couple relationship	150/670	22.3	168/920	18.3	95/670	14.2	98/920	10.6
	≥2 couple relationships	159/410	38.7	144/411	35.0	131/410	32.0	89/411	21.7
Number of sexual partners during the last 5 years	Never had sex	2/17	12.1	5/57	8.6	0/17	0.0	0/57	0.0
	None	34/114	29.8	40/244	16.5	31/114	26.7	27/244	10.9
	One	154/796	19.4	212/1004	21.1	86/796	10.9	111/1004	11.0
	Two or more	377/476	79.2	206/284	72.3	357/476	75.1	182/284	64.2
Self-perceived sexual orientation	Heterosexual	522/1336	39.1	454/1545	29.4	436/1336	32.6	313/1545	20.3
	Bisexual	8/13	62.9	6/11	51.9	8/13	62.9	6/11	51.9
	Homosexual	35/40	87.4	2/8	24.5	28/40	70.7	0/8	0.0
	Not answered	2/14	15.5	2/26	5.8	2/14	14.8	1/26	4.7
Variables related to communication about sexuality and perceptions of HIV									
Intimate social network	0 person	124/557	22.3	77/546	14.1	94/557	16.9	31/546	5.7
	1–2 person(s)	179/375	47.7	167/525	31.8	140/375	37.4	127/525	24.2
	3–5 persons	131/237	55.3	143/288	49.6	121/237	50.9	110/288	38.2
	6–25 persons	60/73	81.8	23/64	36.2	57/73	77.6	17/64	25.9
	Anyone	40/66	60.4	24/59	40.1	35/66	53.4	14/59	24.3
	Not answered	33/94	35.3	29/107	27.5	27/94	28.5	21/107	19.6
Perceived susceptibility to HIV infection	No	350/1054	33.2	263/1221	21.6	290/1054	27.6	171/1221	14.0
	Yes	217/349	62.3	200/369	54.2	184/349	52.7	149/369	40.4
Stigma toward HIV+ people	No	269/520	51.7	243/641	37.8	233/520	44.7	180/641	28.1
	Yes	298/883	33.8	220/948	23.3	242/883	27.4	140/948	14.8
Variables related to HIV prevention behaviours									
Use of condoms	Never/unknown	18/248	7.4	45/524	8.6	0/248	0.1	4/524	0.7
	>5 years ago	81/502	16.1	100/520	19.2	45/502	8.9	42/520	8.1
	≤5 years ago	468/654	71.7	318/545	58.3	429/654	65.7	274/545	50.3
Voluntary HIV testing	No	322/943	34.1	193/1123	17.2	255/943	27.1	127/1123	11.3
	Yes	246/460	53.5	270/467	57.8	219/460	47.7	193/467	41.3

The intimate social network was defined by the number of persons to whom individuals speak about sexuality. Regarding stigma towards HIV+ people, we distinguished participants expressing no stigma at all (i.e. who would accept working with a HIV+ person, having a meal at his/her home, going on leave with him/her, leaving him/her looking after his/her children or grand-children, and having sex with him/her using a condom) from those expressing stigma (i.e. who would not accept at least one of the situations listed above). The perceived susceptibility to HIV infection was asked by a single Yes/No question: “Have you ever feared being infected by AIDS virus?” The questionnaire was based on previous studies [28–31, 34, 35] and pre-tested.

### Statistical analyses

Analyses were performed on 2994 persons, excluding HIV positive people (*n* = 8, as declared either in an open list of “serious and/or chronic” medical conditions, or in the complete list of the chronic conditions fully covered by the French Social security health insurance, in two other parts of the SIRS questionnaire) and non-response (*n* = 4). First, we examined the prevalence of the intention of protection against HIV and the adoption of a condom-based approach for protection against HIV by country of origin and according to all the covariates (Table 1). All the numbers and proportions presented in Table 1 were weighted to account for the complex sample design and for the post-stratification adjustment on age and gender, according to the 2006 census data in Paris area, as described elsewhere [29].

To explore factors associated with the intention of protection and the adoption of a condom-based approach for protection against HIV, we estimated logistic regression models, taking into account the cluster effect (*vce(cluster)*) in Stata 11® software, separately for men and women (as sexual biographies, attitudes and behaviours usually differ between men and women [19, 36]). After estimating the association between migration origin and both outcomes with participants’ age as the only covariables (which was preferred to an univariate model since age influences strongly perceptions and behaviours related to sex), we estimated five models, adding successively variables related to 1) demographics, 2) socioeconomic status, 3) sexual biography, 4) communication about sexuality and perception of HIV, and 5) HIV prevention behaviours. Only covariables significantly associated with at least one outcome (in men or in women) were retained at each step. For simplicity of presentation, only the first models (adjusted on age only) and the final ones are presented here (respectively named model 1 and model 2 in Tables 2 and 3) but all intermediate models can be found in Additional file 1.

**Table 2:** Factors associated with the intention of protection against HIV according to the sex, Paris metropolitan area, France, 2010

		Men				Women			
Variable	Category	Model 1^a^		Model 2^b^		Model 1^a^		Model 2^b^	
		aOR	95 % CI	aOR	95 % CI	aOR	95 % CI	aOR	95 % CI
Migration origin	French with Fr. parents (ref.)	1	-	1	-	1	-	1	-
	French of Sub-Saharan origin	1.39	[0.80,2.41]	2.66	[1.34,5.31]**	0.94	[0.56,1.56]	1.62	[0.84,3.10]
	Sub-Saharan foreigner	1.48	[0.71,3.08]	3.43	[1.66,7.13]***	1.50	[0.93,2.43]	2.94	[1.65,5.23]***
	French of Maghrebi origin	0.63	[0.34,1.17]	1.58	[0.79,3.16]	0.35	[0.22,0.54]***	0.77	[0.42,1.39]
	Maghrebi foreigner	0.81	[0.45,1.45]	1.49	[0.54,4.10]	0.22	[0.10,0.52]***	0.57	[0.23,1.43]
	French of European origin	0.89	[0.44,1.81]	1.08	[0.51,2.25]	0.69	[0.43,1.10]	0.97	[0.55,1.69]
	European foreigner	0.49	[0.26,0.93]*	1.77	[0.86,3.66]	0.58	[0.34,1.00]*	1.02	[0.52,2.03]
	French of other origin	0.87	[0.53,1.42]	1.88	[0.83,4.24]	0.80	[0.53,1.22]	1.37	[0.83,2.25]
	Other foreigner	0.38	[0.17,0.86]*	0.99	[0.25,3.95]	0.15	[0.06,0.41]***	0.46	[0.16,1.28]
Religious affiliation and practice	Regular religious practice			1.04	[0.56,1.95]			1.02	[0.66,1.57]
	Irregular religious practice			1.15	[0.73,1.82]			0.90	[0.64,1.28]
	Relig. affiliation, no practice			0.98	[0.65,1.50]			1.00	[0.70,1.42]
	Neither practice nor affil (ref)			1	-			1	-
Couple status	No relationship			3.06	[1.84,5.08]***			3.30	[2.17,5.01]***
	Love affair			2.04	[1.15,3.59]*			3.00	[1.95,4.63]***
	Non-cohabiting couple			3.65	[1.73,7.69]***			1.49	[0.79,2.83]
	Cohabiting couple (ref.)			1	-			1	-
Lifetime number of couple relationships	Never any couple relationship			2.44	[1.39,4.29]**			2.63	[1.70,4.09]***
	One couple relationship (ref.)			1	-			1	-
	≥2 couple relationships			1.08	[0.76,1.54]			1.44	[1.03,2.03]*
Number of sexual partners duringthe last 5 years	Never had sex			0.40	[0.03,5.59]			0.11	[0.03,0.45]**
	None			1.47	[0.72,3.00]			0.85	[0.53,1.35]
	One			1	-			1	-
	Two or more			1.95	[1.26,3.03]**			2.05	[1.44,2.92]***
Self-perceived sexual orientation	Heterosexual (ref.)			1	-			1	-
	Bisexual			0.49	[0.19,1.24]			0.54	[0.28,1.06]
	Homosexual			2.28	[0.48,10.8]			0.37	[0.07,1.91]
	Not answered			0.22	[0.07,0.71]*			0.69	[0.25,1.86]
Intimate social network	0 person (ref.)			1	-			1	-
	1–2 person(s)			1.54	[1.02,2.34]*			0.90	[0.67,1.20]
	3–5 persons			1.94	[1.21,3.11]**			1.53	[1.05,2.22]*
	6–25 persons			5.15	[2.34,11.3]***			0.91	[0.48,1.71]
	Anyone			2.95	[1.32,6.58]**			2.09	[0.85,5.15]
	Not answered			1.31	[0.64,2.68]			0.99	[0.59,1.68]
Perceived susceptibility to HIV infection	No (ref.)			1	-			1	-
	Yes			1.98	[1.18,3.33]**			1.55	[1.07,2.22]*
Stigma toward HIV+ people	No (ref.)			1	-			1	-
	A bit - a lot			0.63	[0.44,0.91]*			0.70	[0.55,0.89]**
Use of condoms	Never/unknown (ref.)			1	-			1	-
	>5 years ago			2.86	[1.62,5.05]***			1.84	[1.17,2.90]**
	≤5 years ago			17.8	[8.83,35.9]***			4.34	[2.73,6.91]***
Voluntary HIV testing	No (ref.)			1	-			1	-
	Yes			0.58	[0.40,0.83]**			1.90	[1.44,2.52]***

* *p <* 0.05; ** *p <* 0.01; *** *p <* 0.001

^a^: model adjusted on age

^b^: model adjusted on age, level of education and monthly household income per consumption unit

**Table 3:** Factors associated with the adoption of a condom-based approach for protection against HIV according to sex, Paris metropolitan area, France, 2010

		Men				Women			
Variable	Category	Model 1^a^		Model 2^b^		Model 1^a^		Model 2^b^	
		aOR	95 % CI	aOR	95 % CI	aOR	95 % CI	aOR	95 % CI
Migration origin	French with Fr. parents (ref.)	1	-	1	-	1	-	1	-
	French of Sub-Saharan origin	0.65	[0.28,1.51]	0.79	[0.22,2.81]	0.75	[0.41,1.38]	1.62	[0.68,3.88]
	Sub-Saharan foreigner	0.59	[0.23,1.55]	1.38	[0.38,4.93]	0.64	[0.35,1.16]	0.93	[0.40,2.18]
	French of Maghrebi origin	0.39	[0.19,0.79]**	0.75	[0.34,1.67]	0.28	[0.14,0.53]***	0.65	[0.30,1.42]
	Maghrebi foreigner	0.58	[0.32,1.02]	1.15	[0.45,2.95]	0.31	[0.14,0.69]**	0.81	[0.23,2.87]
	French of European origin	1.21	[0.56,2.62]	2.00	[0.84,4.75]	0.64	[0.36,1.13]	1.13	[0.52,2.46]
	European foreigner	0.48	[0.23,1.01]	1.73	[0.58,5.12]	0.47	[0.24,0.92]*	0.93	[0.31,2.82]
	French of other origin	0.58	[0.32,1.07]	0.89	[0.35,2.26]	0.73	[0.42,1.27]	1.28	[0.62,2.63]
	Other foreigner	0.25	[0.10,0.61]**	0.68	[0.16,2.91]	0.32	[0.13,0.82]*	2.34	[0.78,7.00]
Religious affiliation and practice	Regular religious practice			0.93	[0.51,1.71]			0.67	[0.38,1.18]
	Irregular religious practice			1.33	[0.81,2.17]			0.86	[0.51,1.44]
	Relig. affiliation, no practice			0.89	[0.52,1.54]			0.57	[0.40,0.82]**
	Neither practice nor affil (ref)			1	-			1	-
Couple status	No relationship			8.18	[4.46,15.0]***			14.5	[8.35,25.2]***
	Love affair			4.01	[1.91,8.45]***			9.94	[5.57,17.7]***
	Non-cohabiting couple			5.80	[2.43,13.8]***			4.32	[1.96,9.51]***
	Cohabiting couple (ref.)			1	-			1	-
Lifetime number of couple relationships	Never any couple relationship			2.24	[1.16,4.35]*			1.97	[1.31,2.96]**
	One couple relationship (ref.)			1	-			1	-
	≥2 couple relationships			1.14	[0.68,1.90]			1.47	[1.01,2.13]*
Number of sexual partners during the last 5 years	Never had sex			n. e.				n.e.	
	No partner since 5 years			0.81	[0.37,1.76]			0.64	[0.36,1.13]
	One partner last 5 years (ref.)			1	-			1	-
	≥2 partners last 5 years			6.48	[4.17,10.1]***			4.37	[3.04,6.28]***
Self-perceived sexual orientation	Heterosexual (ref.)			1	-			1	-
	Bisexual			0.64	[0.18,2.26]			0.88	[0.40,1.95]
	Homosexual			0.94	[0.29,3.08]			n.e.	
	Not answered			0.16	[0.04,0.59]**			1.15	[0.42,3.16]
Intimate social network	0 person (ref.)			1	-			1	-
	1–2 person(s)			1.37	[0.83,2.28]			1.37	[0.83,2.27]
	3–5 persons			2.60	[1.25,5.39]*			2.39	[1.42,4.00]***
	6–25 persons			3.85	[1.46,10.1]**			1.78	[0.80,3.98]
	Anyone			1.40	[0.66,2.97]			1.67	[0.75,3.73]
	Not answered			0.90	[0.36,2.28]			1.48	[0.68,3.20]
Perceived susceptibility to HIV infection	No (ref.)			1	-			1	-
	Yes			1.42	[0.86,2.34]			1.46	[1.03,2.05]*
Stigma toward HIV+ people	No (ref.)			1	-			1	-
	A bit - a lot			0.63	[0.42,0.95]*			0.51	[0.36,0.72]***
Voluntary HIV testing	Never/unknown (ref.)			1	-			1	-
	>5 years ago			1.10	[0.72,1.70]			1.67	[1.11,2.51]*

*ne* not estimated (no individual in the category has adopted a condom-based approach for protection against HIV)

* *p <* 0.05; ** *p <* 0.01; *** *p <* 0.001

^a^: model adjusted on age

^b^: model adjusted on age, level of education and monthly household income per consumption unit

Finally, we conducted a mediation analysis in order to disentangle effects of stigma and perceived susceptibility to HIV infection on the intention of protection against HIV.

## Results

### Individual characteristics

Altogether 87.4 % of the study population was French, 3.2 % was Sub-Saharan African and 2.9 % was Maghrebi (Table 1). Among the French citizens, 24.7 % were of foreign origin according to our definition; this included migrants who acquired French citizenship (53.6 % of French with foreign origins) together with French-born, non-migrants who were born in France from foreign parents.

### Intention of protection

About one third of the sample (31.0 %) stated that they protected themselves from HIV (Table 1). Men were more likely to do so than women (36.8 vs. 27.3 %, *p <* 0.001). Only 4 in 3006 interviewees (0.1 %) didn’t answer that question.

Being a foreigner from Europe or other parts of the world except Africa was associated with a less frequent intention of protection in men (aOR = 0.49 and aOR = 0.38 respectively, Table 2, men, model 1) and women (aOR = 0.28 and aOR = 0.15 respectively, Table 2, women, model 1), but these differences were no longer significant in the fully adjusted models (Table 2, models 2). Conversely, we observed that, in men, the addition of socioeconomic variables revealed strong and significant associations between being French of SSA origin or being foreigner from SSA and the intention of protection, that persisted in the full model, with aOR increasing up to 2.66 (95 % CI = [1.34–5.31]) and 3.43 (95 % CI = [1.66–7.13]), respectively (Table 2, men, model 2). We observed a similar result in women, but for SSA foreigners only with aOR increasing up to 2.94 (95 % CI = [1.65–5.23], Table 2, women, model 2).

French women of Maghrebi origin and foreign Maghrebi women were both less likely to declare an intention of protection against HIV than the majority population when adjusting for age (aOR = 0.35 and aOR = 0.22 respectively, Table 2, women, model 1) but these two associations were no longer statistically significant when adjusting for the others variables (Table 2, women, model 2). No significant differences were ever observed for the corresponding male populations.

In univariate analysis, people who practice a religion were less likely to declare protecting themselves from HIV (OR of regular practice for both sex = 0.68, 95 % CI = [0.56–0.84]) but this association disappeared when variables related to the sexual biographies were added to the model (Table 2, models 2).

Interestingly, the association between voluntary HIV testing and the intention of protection differed notably between genders in multivariate analysis. In women, testing was associated with more intention of protection (aOR = 1.90, 95 % CI = [1.44–2.52], Table 2, women, model 2) while men having been voluntarily tested for HIV expressed less intention to protect themselves (aOR = 0.58, 95 % CI = [0.40–0.83], Table 2, men, model 2). Additionally, the strength of the associations between condom use or intimate social network and the intention of protection were more important in men than in women.

### Adoption of a condom-based approach for protection against HIV

Among the 2994 HIV negative people who had answered the question about the intention of protection, 680 (22.7 %) declared using condoms steadily to protect themselves from HIV (Table 1), more often men (29.5 %) than women (18.3 %, *p <* 0.001). Most of those who chose condoms as a means of protection had expressed an intention of protection (26/688, 96.2 %) but, inversely, 29.3 % (275/937) of people who intended to protect themselves from HIV selected other means than condom use.

In men, being French of Maghrebi origin or foreign from out of Africa and Europe was associated with less recourse to a condom-based approach of protection when adjusted for age (aOR = 0.39 and aOR = 0.25 respectively, Table 3, men, model 1). These associations disappeared when we adjusted for variables regarding sexual biographies (Table 3, men, model 2). No significant association with a SSA origin or nationality was observed.

In women, in the age-adjusted models (Table 3, women, model 1), being French of Maghrebi origin and being a Maghrebi foreigner were both associated with a lower probability of declaring a condom-based approach for protection: aOR = 0.28, 95 % CI = [0.14–0.53] and aOR = 0.31, 95 % CI = [0.14–0.69], respectively. It was also the case for foreign women from Europe or other parts of the world except for Africa (aOR = 0.47 and aOR = 0.32 respectively) but, as in men, these differences were no longer significant in the fully adjusted models (Table 3, women, model 2).

Globally, adjustment variables were associated with the recourse to a condom-based approach of protection in the same way than they were with the intention of protection, except that we did not observe here any significant association with the perceived susceptibility to HIV infection, nor with voluntary HIV testing, among men.

## Discussion

Our results show that people’s HIV prevention attitudes differ according to their country of origin. First, models adjusting only for demographic features show that Maghrebi women and French women of Maghrebi origin try less to protect themselves from HIV, and rely less on condom for protection against HIV than French women with French parents. This result suggests that Maghrebi women are insufficiently reached by prevention campaigns. It may be because they have a limited access to the messages, e.g. because they are not an actual target group in a context where most new HIV infections in foreign women are diagnosed in women originating from Sub-Saharan Africa. It may also be due to messages being insufficiently tailored for these persons, that are then not clearly understood or don’t translate into a change in attitude or behaviour, e.g. because of the women’s status in some of the Maghrebi immigrant subgroups that hinders communication about sexuality or because they distance themselves from risk groups. As noted already 10 years ago in France [27], Maghrebi population –originally from countries where the HIV epidemic is concentrated in high-risk groups [37] – still miss prevention interventions as compared to the rest of the general population in France. This may result from a low level of acculturation in France, which is a barrier to health-related communication and specifically prevention messages [38] and deserve the reinforcement of specific, targeted, information and prevention programs. These associations between Maghrebi citizenship or origin and a reduced intention for protection against HIV disappear when variables reflecting the women’s socioeconomic situation and sexual biography are included into the models: it seems that these characteristics outweigh migration origin and/or – at least partially - explain the origin-related differences previously observed.

A very similar situation was observed among the few foreign women from non-African and non-European countries included in our sample. The small number of these women (a heterogeneous population structured in small communities in the Parisian population) and, also, the fact that our sample missed the neighbourhoods inhabited by Asian communities (although numerous in the Parisian area) make this result difficult to interpret and infer.

The second foremost result is that, in fully adjusted models, foreigners from SSA and French men of SSA origin declare more often that they do something to protect themselves from HIV than the French with French parents. This can be due to recent prevention campaigns specifically addressed to SSA populations in France (and/or those in their countries of origin) [13, 15] since perceiving oneself as being part of a risk group influence prevention behaviours [21, 22]. It can also be related to certain forms of positive discrimination in individual HIV prevention promotion performed by health care workers, similarly to what has been previously described for HIV testing [22, 26]. Alternatively it may be related to a social desirability bias that is known to be more frequent in cultures based on oral tradition such as those in SSA [39]. It is worth noting that this more frequent declaration of protection did not translate into a more frequent recourse to the use of condoms as a means of protection in any of these 3 subgroups. If, generally speaking, most of the individual characteristics associated with the intention to protect oneself against HIV were also associated with a condom-based approach, this was less true for the origin from SSA, possibly because people in this category opt more for other means such as HIV testing, as described in the Paris area [40]. Concerning women, it has been shown that they are used to resort to other means of protection than condoms only [34]. These various results confirm the need for a culture-specific approach of HIV prevention and in the interpretation of results of HIV related surveys [11, 22, 24, 26, 40].

Globally, our results show that French people with foreign origin are in an intermediate situation between French people with French parents and foreigners from the same continent. This likely reflects the acculturation process [4] and, more precisely, the maintenance – or the re-appropriation - of cultural norms inherited from the culture of origin. Given the definition of the different categories of French people with foreign origins used here, we cannot go further in this cultural interpretation and distinguish between the situation of people born French in France to foreign parents and those of the immigrants who acquired the French citizenship (each representing about half of the above mentioned categories). Conversely, even if limited in their interpretation, our results are the first quantitative results ever published in France in a representative, population-based sample about the situation of the French people of foreign origin in the field of HIV prevention (and of HIV epidemiology in general). We think that replicating such approaches in other European countries would be very useful at the time when European public health institutions, such as the European Centre for Disease Prevention and Control, emphasize that prevention efforts should not only aim at the general population but also at groups such as immigrants or minorities [41, 42]. Their results may usefully precede and/or trigger the development of HIV prevention interventions targeted to immigrants at a larger scale or with more significant resources.

Looking at covariables, some results deserve being discussed as they confirm or infirm previous findings. We found that people with a religious affiliation were less likely to intend for protecting themselves against HIV, similarly to what has been observed previously in France [40, 43] but this influence was not observable when we adjusted for variables related to sexual biography. This suggests that the impact of religion on means of prevention may be limited to (or mediated by) an influence on the sexual biography even if further analysis would be necessary to explore this pathway. Conversely, our results clearly show that affective and marital status has a major impact on the individual’s definition of protection means for oneself and plea, with others, for a systematic adjustment for the couple status to study these HIV prevention strategies, which was not done in the above mentioned study [43]. We also observed that individuals stigmatizing HIV+ people were less likely to intend to protect themselves against HIV and to opt for condom-based approaches for protection. This result was expected since self-protective attitudes and stigma share common determinants such as perceived severity of AIDS and perceived self-susceptibility to HIV infection [44]. Nevertheless the fact that the stigma and the perceived susceptibility to HIV infection are both independent factors in our models and a restricted mediation analysis (not presented) showing that only 12.3 % of the effect of stigma on the intention of protection is mediated by the perceived susceptibility, suggest that stigma and perceived susceptibility are related with intended prevention behaviours mostly through unconnected pathways, unlike suggested elsewhere [45]. The importance of other determinants, such as stereotypes, has not been evaluated in our study and it is difficult to do so in a quantitative study like this one. Obviously, qualitative studies make invaluable contributions to a deep and comprehensive knowledge of the various determinants of these attitudes and behaviours [11].

This study presents some limitations. Our sample is restricted to Paris metropolitan area and our results cannot be extrapolated to the whole France. However since this region bears the most important part of the HIV burden in mainland France, our conclusions are pertinent to help in the management of the epidemic in France but also in foreign settings with similar features [46]. The attrition rate between 2005 and 2010 might have influenced the representativeness of the sample, but has few chance to affect the relationships between variables in a sample of this size [47] and we must remind here that the non-reinterviewed people have been replaced by people selected by the same random procedure (with a quite good response rate for a population-based survey). Also the sampling design didn’t allow for reaching very vulnerable populations such as homeless people, among which immigrants are overrepresented [48] and we interviewed only people speaking French when non-French speaking people accounted for 6.8 % of individuals contacted for the study. If those people could have been included and interviewed in SIRS, the difference between French people and foreigners might have been accentuated (by construction, our sample does not allow us either to test the influence of speaking French or not). The questionnaire of the SIRS surveys didn’t allow for the investigation of ways of transmission of HIV other than sexual and was also limited by the measurement of multipartnership over the last 5 years only, while it is usually calculated on the sexual activity of the previous year [19, 49]. Also, several factors known to impact the strategies of protection against STDs were not measured, such as the social context in which a sexual relationship takes place [50]. Moreover, since our questions investigated attitudes and declared behaviours but not practices (e.g. the utilization of condoms during last sexual intercourses or according to different kinds of sexual partners), we are unable to evaluate the gap between intention and effective practices [19].

## Conclusion

According to their migration origin, some foreigners and French people of foreign origin show attitudes and behaviours profiles regarding HIV protection that are different from those of the majority of the population in France. These differences argue for the importance of a culture-specific approach of HIV prevention. Even if some attention is currently paid for immigrants from SSA, our results draw attention to other fractions of the population that could still escape from prevention messages in France, especially people with Maghrebi origins. In this way, our results plea for further investigations (and a public health monitoring) of their situations in France.

## Additional file

Additional file 1:
**Complete intermediate multivariate models.**
